# On Conformal Conic Mappings of Spherical Domains

**DOI:** 10.1155/2014/840280

**Published:** 2014-01-14

**Authors:** Andrei Bourchtein, Ludmila Bourchtein

**Affiliations:** Institute of Physics and Mathematics, Pelotas State University, Brazil

## Abstract

The problem of the generation of homogeneous grids for spherical domains is considered in the class of conformal conic mappings. The equivalence between secant and tangent projections is shown and splitting the set of conformal conic mappings into equivalence classes is presented. The problem of minimization of the mapping factor variation is solved in the class of conformal conic mappings. Obtained results can be used in applied sciences, such as geophysical fluid dynamics and cartography, where the flattening of the Earth surface is required.

## 1. Introduction

The problem of the generation of homogeneous grids for spherical domains is one of the oldest problems of cartography and geodesy, and it is also the important part of developing efficient numerical schemes for geophysical simulations, in particular, for atmosphere-ocean dynamics. Modeling large-scale atmosphere-ocean processes implies the use of the spherical geometry for the formulation of the governing equations. Computational grids based on the spherical coordinates are highly nonhomogeneous that cause the problems for both dynamical and physical parts of numerical schemes [1–3]. The most efficient way to circumvent this problem is an application of conformal mappings from a sphere onto a plane, because these transformations usually maintain a simpler form of the governing equations and also assure local isotropy and smoothness of variation of the physical mesh sizes on computational grids [1–6].

Each conformal mapping can be characterized by its mapping factor *m* representing the ratio between elementary arc lengths along a projective curve (image) and corresponding spherical curve (original). If a physical problem requires the use of the physical space mesh size *h*_0_, then the ideal grid is physically homogeneous with the same mesh size *h*_0_ over the entire domain. On homogeneous computational grid, the physical mesh size usually varies providing better actual (physical) approximation in the regions where the mapping factor *m* has the maximum values (*m*_max_) and worse approximation in the regions with the minimum mapping factor (*m*_min_). As a measure of the homogeneity of the computational grid one can use the ratio between the maximum and minimum values of the mapping factor over the considered domain:

(1) $$\begin{array}{c} \alpha =\frac{m_{\max}}{m_{\min}}. \end{array}$$

In particular, this criterion is suitable for generation of computational grids for explicit and semi-implicit schemes [1, 7, 8]. As far as we know, the use of the variation coefficient *α* for measuring the homogeneity of the computational grids was first proposed and studied in [1] and the further analysis of the properties of this coefficient and justification of its application in the atmosphere-ocean numerical models was performed in different works of the same authors (e.g., [4, 7, 8]).

Thus, the problem of computational grid optimization can be formulated as a search for the mapping of the spherical domain that assures the minimum values of the variation coefficient *α* over the considered spherical domain *Ω*. In this study, we consider the problem of minimization of *α* in the class of conic mappings, which are standard official cartographic projections for intermediate and large-scale regions of the Earth surface [9–12] and which are frequently used in the modeling of atmosphere and ocean dynamics in the middle and low latitudes [13–20].

## 2. Equivalence Classes of the Conic Mappings

Let us recall the expressions involved in the definition of conic conformal mappings. The formulas of the secant conformal conic projections can be written as follows [9–11]:

(2) ψ=nλ,r=acosφ1n(tan(π/4−φ/2)tan(π/4−φ1/2))n =acosφ2n(tan(π/4−φ/2)tan(π/4−φ2/2))n,m=cosφ1cosφ(tan(π/4−φ/2)tan(π/4−φ1/2))n=cosφ2cosφ(tan(π/4−φ/2)tan(π/4−φ2/2))n,n=ln(cosφ1/cosφ2)ln(tan(π/4−φ1/2)/tan(π/4−φ2/2)),

where *φ*_1_, *φ*_2_ are the secant (standard) latitudes, −*π*/2 < *φ*_1_ < *φ*_2_ < *π*/2, that is, such latitudes where elementary spherical arch length is equal to projection arc length.

The tangent conformal conic mappings have the following form [9–11]:

(3) $$\begin{array}{c} \psi =n\lambda ,\;\;r=a\frac{\cos\varphi_{0}}{n}{\left(\frac{\tan\left(\pi /4-\varphi /2\right)}{\tan\left(\pi /4-\varphi_{0}/2\right)}\right)}^{n}, \end{array}$$

(4) $$\begin{array}{c} m=\frac{\cos\varphi_{0}}{\cos\varphi }{\left(\frac{\tan\left(\pi /4-\varphi /2\right)}{\tan\left(\pi /4-\varphi_{0}/2\right)}\right)}^{n}, \end{array}$$

(5) $$\begin{array}{c} n=\sin\varphi_{0}, \end{array}$$

where *φ*_0_ ∈ (−*π*/2, *π*/2) is the tangent latitude. (Note that the tangent formulas can be obtained from the secant ones by calculating the limit as *φ*_1_ and *φ*_2_ approach *φ*_0_.) Although conformal conic mappings have no exact geometric meaning, the terms secant and tangent are widely used both in cartography and in atmosphere-ocean modeling [3, 5, 9, 10, 12, 21].

We will call two conformal projections equivalent if the ratio between their mapping factors is a constant; that is, the first projection with the mapping factor *m* is equivalent to the second with the mapping factor $\bar{m}$ on domain *Ω* if there exists a constant *k* > 0 such that $m=k\bar{m}$ for any (*λ*, *φ*) ∈ *Ω*. Obviously, equivalent conformal projections have the same space resolution because the transformation from one coordinate system to an equivalent one does not affect physical resolution but only influences the choice of the system of units. The equivalence of two projections implies the equality of their variation coefficients defined by formula (1).

Two conic mappings (secant or tangent) are equivalent on a chosen domain if, and only if, they have the same value of the parameter *n*. In fact, the condition

(6) m=cosφ1cosφ(tan(π/4−φ/2)tan(π/4−φ1/2))n=kcosφ¯1cosφ(tan(π/4−φ/2)tan(π/4−φ¯1/2))n¯=km¯

can be transformed to

(7) (tan(π4−φ2))n−n¯=kcosφ¯1cosφ1(tan(π/4−φ1/2))n(tan(π/4−φ¯1/2))n¯=const 

which implies $n=\bar{n}$. On the other hand, the condition $n=\bar{n}$ results in $m=k\bar{m}$.

Now we will specify the range of variation of the parameter *n*. To this end, we first prove two auxiliary lemmas.

**Lemma 1**

The real-value function

(8) $$\begin{array}{c} f\left(x\right)=x^{n+1}+x^{n-1},\;x\in \left(0,+\infty \right),\;\;n\in \left(0,1\right) \end{array}$$

is

(1) continuous on its domain;
(2) strictly decreasing on the interval (0, *x*_min_) and strictly increasing on the interval (*x*_min_, +*∞*), where
  (9) $$\begin{array}{c} x_{\min}=\sqrt{\frac{1-n}{1+n}}\in \left(0,1\right) \end{array}$$
 is the only minimum point of *f*(*x*);
(3) two-sided unbounded:
  (10) $$\begin{array}{c} \underset{x\to 0^{+}}{\lim}f\left(x\right)=\underset{x\to +\infty }{\lim}f\left(x\right)=+\infty . \end{array}$$

**Proof**

In fact, the property (1) is evident. Moreover, it is clear that *f*(*x*) is an analytic function on its domain. Calculating the first order derivative

(11) f′(x)=(n+1)xn+(n−1)xn−2=xn−2[(n+1)x2+(n−1)]

and observing that *x* ^*n*−2^ > 0, one can obtain the only critical point

(12) $$\begin{array}{c} x_{\text{cr}}=\sqrt{\frac{1-n}{1+n}}, \end{array}$$

which belongs to the interval (0,1). Since the derivative (11) is negative on the interval (0, *x*_cr_) and positive on the interval (*x*_cr_, +*∞*), the property (2) holds.

Finally, the property (3) follows from

(13) limx→0+xn+1=0,  limx→0+xn−1=+∞,limx→+∞xn+1=+∞,  limx→+∞xn−1=0.

The results of Lemma 1 together with the properties of continuous functions (the Intermediate Value Theorem) guarantee that *f*(*x*) takes the same values in exactly two different points *x*_1_, *x*_2_ such that *x*_1_ < *x*_min_ < *x*_2_. The only exception is the minimum point *x*_min_.

It leads to the following.

**Corollary 2**

The equation

(14) $$\begin{array}{c} x^{n+1}+x^{n-1}=c,\;x\in \left(0,+\infty \right),\;\;n\in \left(0,1\right) \end{array}$$

has two solutions if *c* > *f*_min_ has the only solution *x*_min_ if *c* = *f*_min_ and has no solutions if *c* < *f*_min_. Here,

(15) fmin≡f(xmin)=(1−n1+n)n+1+(1−n1+n)n−1=(1+n1−n)(1−n)/2·21+n.

Evidently, *f*_min_ ∈ (1,2) because both factors in the right-hand side of (15) are greater than 1 and *f*(1) = 2.

One can reformulate this corollary in the following way.

**Corollary 3**

The equation

(16) $$\begin{array}{c} x^{n+1}+x^{n-1}=t^{n+1}+t^{n-1},\;0<x<t,\;\;n\in \left(0,1\right) \end{array}$$

has infinite set of solutions (*x*, *t*), where *x* ∈ (0, *x*_min_]. The respective set of *t* values covers the interval [*x*_min_, +*∞*).

Based on this result we can prove the following.

**Lemma 4**

The equation

(17) ln(cosφ1/cosφ2)ln(tan(π/4−φ1/2)/tan(π/4−φ2/2))=n,       −π2<φ1<φ2<π2, n∈(0,1)

has infinite set of solutions (*φ*_1_, *φ*_2_) with any *φ*_2_ from the interval (*φ*_min_, *π*/2], where

(18) $$\begin{array}{c} \varphi_{\min}=\frac{\pi }{2}-2\arctan\sqrt{\frac{1-n}{1+n}},\;0<\varphi_{\min}<\frac{\pi }{2}. \end{array}$$

**Proof**

Equation (17) can be rewritten as follows:

(19) ln((tan(π/4−φ1/2)/(tan2(π/4−φ1/2)+1))tan(π/4−φ2/2)/(tan2(π/4−φ2/2)+1)) ×(ln(tan(π/4−φ1/2)tan(π/4−φ2/2)))−1=n.

Introducing the new variables

(20) $$\begin{array}{c} x_{1}=\tan\left(\frac{\pi }{4}-\frac{\varphi_{2}}{2}\right),\;\;x_{2}=\tan\left(\frac{\pi }{4}-\frac{\varphi_{1}}{2}\right), \end{array}$$

where 0 < *x*_1_ < *x*_2_ < +*∞*, one can reduce (19) to the form

(21) $$\begin{array}{c} \ln\left(\frac{\left(x_{1}/\left(x_{1}^{2}+1\right)\right)}{\left(x_{2}/\left(x_{2}^{2}+1\right)\right)}\right)=n\ln\frac{x_{1}}{x_{2}} \end{array}$$

or

(22) $$\begin{array}{c} x_{1}^{n+1}+x_{1}^{n-1}=x_{2}^{n+1}+x_{2}^{n-1},\;0<x_{1}<x_{2},\;\;n\in \left(0,1\right). \end{array}$$

Thus, (17) is reduced to the equivalent equation (22), which has infinite set of solutions (*x*_1_, *x*_2_) with *x*_1_ ∈ (0, *x*_min_] due to Corollary 3. Therefore, (17) has infinite set of solutions (*φ*_1_, *φ*_2_) with *φ*_2_ ∈ (*φ*_min_, *π*/2], *φ*_min_ = *π*/2 − 2arctan*x*_min_. Besides, 0 < *φ*_min_ < *π*/2 because 0 < *x*_min_ < 1.

Now we can derive the main result about the parameter *n*.

**Theorem 5**

The parameter *n* defined by the formula

(23) n=  ln(cosφ1/cosφ2)ln(tan(π/4−φ1/2)/tan(π/4−φ2/2)),             −π2<φ1<φ2<π2,

belongs to the interval (0,1) if, and only if, the condition *φ*_1_ + *φ*_2_ > 0 is satisfied.

**Proof**

Using the change of variables (20) we rewrite (23) in the form

(24) $$\begin{array}{c} \ln\left(\frac{\left(x_{1}/\left(x_{1}^{2}+1\right)\right)}{\left(x_{2}/\left(x_{2}^{2}+1\right)\right)}\right)=n\ln\frac{x_{1}}{x_{2}} \end{array}$$

with 0 < *x*_1_ < *x*_2_ < +*∞*. The parameter *n* belongs to (0,1) if, and only if,

(25) $$\begin{array}{c} \ln\frac{x_{1}}{x_{2}}<\ln\left(\frac{\left(x_{1}/\left(x_{1}^{2}+1\right)\right)}{\left(x_{2}/\left(x_{2}^{2}+1\right)\right)}\right)\;<0. \end{array}$$

These inequalities are equivalent to

(26) $$\begin{array}{c} \frac{x_{1}}{x_{2}}<\frac{x_{1}}{x_{2}}\cdot \frac{1+x_{2}^{2}}{1+x_{1}^{2}}<1. \end{array}$$

Since 0 < *x*_1_ < *x*_2_, the left inequality is satisfied. The right inequality can be simplified to the equivalent form *x*_1_ · *x*_2_ < 1; that is,

(27) $$\begin{array}{c} \tan\left(\frac{\pi }{4}-\frac{\varphi_{1}}{2}\right)\cdot \tan\left(\frac{\pi }{4}-\frac{\varphi_{2}}{2}\right)<1 \end{array}$$

in the original variables. It can be transformed to the equivalent inequality

(28) $$\begin{array}{c} \sin\frac{\varphi_{1}+\varphi_{2}}{2}>0, \end{array}$$

which holds if, and only if, 0 < *φ*_1_ + *φ*_2_ < *π*.

**Remark 6**

Although conformal mappings have no exact geometric representation, the obtained restriction *φ*_1_ + *φ*_2_ > 0 is the condition of the construction of geometric secant cone with the apex above the North Pole. In many references [3, 5, 10, 21] this condition (or even more restricted condition *φ*_1_ > 0) is implied implicitly as a natural condition for assuring the possibility of projection on a geometric cone located above the North Pole. However, it is worth noting that “geometric point of view” can not be directly applied to conformal conic mappings and, consequently, the result of the proved theorem is not evident for conformal mappings.

**Remark 7**

The last result together with the equivalence condition for conformal conic mappings means that any secant conic projection is equivalent to a certain tangent projection (with the same value of *n*). Moreover, each tangent conic projection with specific value of *n* generates its equivalence class of mappings and all equivalence classes are described by tangent projections when *n* varies on the interval (0,1), that is, for *φ*_0_ ∈ (0, *π*/2). It is interesting to note that this equivalence, which could be “evident” from “geometric point of view,” is not mentioned in the references. Moreover, the statement that secant projections represent a given spherical domain better than tangent ones can be found in various sources [3, 10–12, 21].

**Remark 8**

It can be shown in a similar way that the condition *φ*_1_ + *φ*_2_ < 0 is equivalent to *n* ∈ (−1,0), and it gives rise to conic mappings with “geometric apex” above the South Pole. Each projection of this family has its counterpart among the conic projections with *n* ∈ (0, 1). Therefore, it is sufficient to study only the latter mappings.

Based on the equivalence properties of the conic mappings, we can conclude that the problem of minimization of the variation coefficient *α* in some spherical domain *Ω*  is reduced to the choice of the “best” projection among the tangent conic mappings with *n* ∈ (0,1), or, equivalently, with the tangent latitude *φ*_0_ varying in (0, *π*/2).

## 3. Optimal Choice of Conic Mappings

First we define more precisely spherical domain *Ω*. Since the expression of the mapping factor *m* for conic projections does not depend on the longitude *λ*, the same is true for the variation factors. Therefore, the specification of a domain *Ω*  can be given by its north-south extension. For example, we can define two extremal latitudes *φ*_1_ and *φ*_2_; that is, define the latitude interval $\left[\bar{\varphi }-\gamma ,\bar{\varphi }+\gamma \right]$, where the parameters $\bar{\varphi }=\left(\varphi_{2}+\varphi_{1}\right)/2$ and *γ* = (*φ*_2_ − *φ*_1_)/2 determine the domain location and size with respect to latitude. Note that any conic projection with *n* ∈ (0, 1) neither is defined at the South Pole nor has the mapping factor defined at the North Pole. Therefore, the interval [*φ*_1_, *φ*_2_] must be located inside the open interval (−*π*/2, *π*/2); that is, −*π*/2 < *φ*_1_ < *φ*_2_ < *π*/2, with *φ*_1_ + *φ*_2_ > 0. This implies that $\bar{\varphi }\in (0,\pi /2)$ and $\gamma <\pi /2-\bar{\varphi }$.

Now we can prove the following minimization theorem.

**Theorem 9**

The minimum variation of the mapping factor (4) is attained at the latitude *φ*_opt_ defined by

(29) n=sinφopt=ln cos φ1−ln cos φ2ln tan(π/4−φ1/2)−ln tan(π/4−φ2/2).

For this *n* the variation coefficient is expressed as follows:

(30) α=cosφoptcosφ2(tan(π/4−φ2/2)tan(π/4−φopt/2))sinφopt=cosφoptcosφ1(tan(π/4−φ1/2)tan(π/4−φopt/2))sinφopt.

**Proof**

First we show that for any fixed *φ*_0_ ∈ (0, *π*/2) the positive function

(31) m(φ,φ0)=cosφ0cosφ(tan(π/4−φ/2)tan(π/4−φ0/2))sinφ0,   φ∈[φ1,φ2], φ1+φ2>0,

has the absolute minimum value equal to 1 at the point *φ*_0_ and the absolute maximum value at least at one of the end points of the interval [*φ*_1_, *φ*_2_].

To this end, let us consider the auxiliary function

(32) f(φ)=2cosφ(tan(π4−φ2))n=(tan(π4−φ2))n−1·(tan2(π4−φ2)+1),    φ∈  [φ1,φ2], n=sinφ0∈(0,1).

Changing the independent variable by the formula *x* = tan(*π*/4 − *φ*/2), we can rewrite (32) as follows:

(33) f(x)=xn+1+xn−1, x∈[x1,x2],  n∈(0,1),x1=tan(π4−φ22),  x2=tan(π4−φ12).

By Lemma 1, the function (33) attains the absolute minimum value at the point

(34) $$\begin{array}{c} x_{\min}=\sqrt{\frac{1-n}{1+n}} \end{array}$$

and the absolute maximum value at one or both of the end points of the interval [*x*_1_, *x*_2_]. This means that the absolute minimum point of the function (32) is *φ*_min_ = *φ*_0_, because

(35) $$\begin{array}{c} \tan\left(\frac{\pi }{4}-\frac{\varphi_{\min}}{2}\right)=\sqrt{\frac{1-n}{1+n}}=\tan\left(\frac{\pi }{4}-\frac{\varphi_{0}}{2}\right) \end{array}$$

and the absolute maximum point is *φ*_1_ or *φ*_2_. Therefore, the same result is true for the original function (31), and substituting *φ*_0_ instead of *φ* in this function we obtain that *m*_min_(*φ*_0_) = *m*(*φ*_0_, *φ*_0_) = 1. Hence,

(36) $$\begin{array}{c} \alpha \left(\varphi_{0}\right)=m_{\max}\left(\varphi_{0}\right)=\max\left\{m\left(\varphi_{1},\varphi_{0}\right),m\left(\varphi_{2},\varphi_{0}\right)\right\}. \end{array}$$

Now we should minimize the function (36) with respect to the parameter *φ*_0_. Obviously, the solution of this problem is found from the condition *m*(*φ*_1_, *φ*_0_) = *m*(*φ*_2_, *φ*_0_); that is,

(37) cosφ0cosφ2(tan(π/4−φ2/2)tan(π/4−φ0/2))sinφ0  =cosφ0cosφ1(tan(π/4−φ1/2)tan(π/4−φ0/2))sinφ0.

Simplifying this equation and solving with respect to *n* = sin *φ*_0_, we obtain formula (29).

**Remark 10**

**Figure 1:**
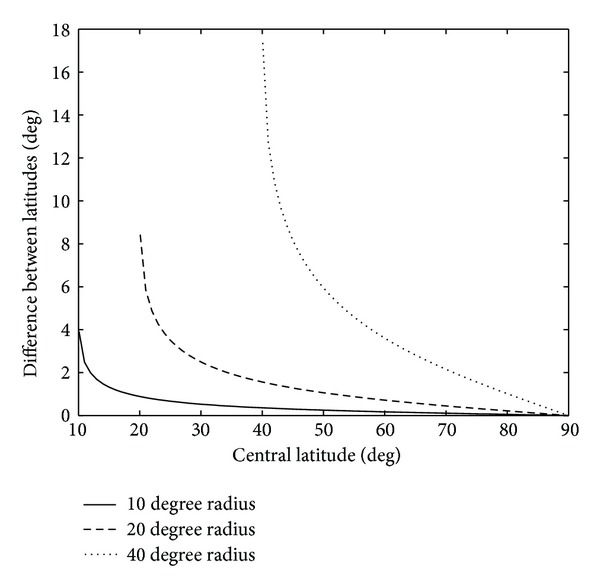
Differences between the optimal tangent latitude and center latitude for different domains.

Note that formula (29) defines the values of the optimal tangent latitude in the interval $\left(\bar{\varphi },\varphi_{2}\right)$. The difference between *φ*_opt_ and $\bar{\varphi }$ increases with approximation to the North Pole and with increase of the radius *γ*. In Figure 1 these differences are shown as functions of the centerpoint $\bar{\varphi }\;$for different values of *γ*.
